# Comparing two interdisciplinary occupational rehabilitation programs for employees on sick leave: a mixed-method design study protocol

**DOI:** 10.1186/s12891-021-03994-3

**Published:** 2021-02-09

**Authors:** Monica Eftedal, Torill H. Tveito, Ulrik Gensby, M. Kamrul Islam, Stein Atle Lie, Gro Aasland, Svein Kostveit, Chris Jensen

**Affiliations:** 1 National Advisory Unit on Occupational Rehabilitation, Haddlandsvegen 20, 3864 Rauland, Norway; 2 NORCE Norwegian Research Centre, Bergen, Norway; 3 Department of Health, Social and Welfare Studies, University of South-Eastern Norway, Horten, Norway; 4 Team Working Life Aps, Copenhagen, Denmark; 5 Adjunct Scientist, Institute for Work and Health, Toronto, Canada; 6 Department of Public Health and General Practice, Norwegian University of Science and Technology, Trondheim, Norway; 7 Department of Clinical Dentistry, University of Bergen, Bergen, Norway; 8 Division of Physical Medicine and Rehabilitation, Vestfold Hospital Trust, Tønsberg, Norway; 9 The Rehabilitation Centre AiR as, Rauland, Norway

**Keywords:** Study protocol, Mixed method, Return to work, Occupational rehabilitation, Musculoskeletal disorders, Common mental disorders, Process evaluation

## Abstract

**Background:**

Musculoskeletal disorders (MSDs) and common mental disorders (CMDs) are the most frequent reasons for long-term sick leave and work disability. Occupational rehabilitation programs are used to help employees return to work (RTW). However, knowledge regarding the effect of these programs is scarce, and even less is known about which programs are best suited for which patients. This study aims to compare the RTW results of two interdisciplinary occupational rehabilitation programs in Norway, as well as to examine the delivery and reception of the two programs and explore the active mechanisms of the participants’ RTW processes.

**Methods/design:**

We will use a mixed-method convergent design to study the main outcome. Approximately 600 participants will be included in the study. Eligible study participants will be aged 18–60 years old and have been on sick leave due to MSDs, CMDs, or both for at least 6 weeks. Interdisciplinary teams at both participating clinics will deliver complex occupational rehabilitation programs. The inpatient rehabilitation program has a duration of 4 weeks and is full time. The outpatient program has a duration of 3 months and involves weekly sessions. The primary outcome is RTW. Secondary outcomes are differences in the incremental cost for an averted sick leave day, cost utility/benefit, and differences between the programs regarding improvements in known modifiable obstacles to RTW. Subgroup analyses are planned. The researchers will be blinded to the intervention groups when analyzing the quantitative RTW data.

**Discussion:**

This study aims to provide new insights regarding occupational rehabilitation interventions, treatment targets, and outcomes for different subgroups of sick-listed employees and to inform discussions on the active working mechanisms of occupational rehabilitation and the influence of context in the return-to-work process.

**Trial registration:**

Current controlled trials ISRCTN12033424, 15.10.2014, retrospectively registered.

## Background

Work disability represents a huge problem not only for employees, but also for their families, workplaces, and society [1]. At the end of 2017, a total of 16.9% of the Norwegian population between 18 and 66 years old received a health-related benefit [2]. In the second quarter of 2020, the total rate of physician-certified sickness absence was 5.2%; 32.2% of the absences were due to musculoskeletal disorders (MSDs), and 17.3% were due to common mental disorders (CMDs) [3]. Comorbidities are also common and are associated with an increased length of sick leave and increased rates of disability pension [4, 5]. General practitioners in Norway use referrals to complex occupational rehabilitation programs in specialist health services for some of these patients to promote faster and more sustainable RTW. However, the path towards RTW is a dynamic process [6], and the outcome cannot be accurately predicted just from knowledge of the medical or physical dimensions of the disorder or condition [7]. At the same time, knowledge regarding the effects of these occupational programs and who will benefit from which intervention is insufficient. In general, more studies are needed to evaluate interventions both for patients with MSDs [8] and those with CMDs [9]. Additionally, studies with a process evaluation are needed in order to improve our knowledge about intervention programs, the RTW process, and the influence of context [10]. Since no treatment is likely to affect all patients with the same disorder in the same way [11], more subgroup analyses in evaluations of occupational rehabilitation programs are also recommened [12].

In this project, we aim to increase the knowledge in this area by evaluating two occupational rehabilitation interventions in Norway and their treatment targets and outcomes for different subgroups of sick-listed employees and to inform discussions on active working mechanisms of occupational rehabilitation and the influence of context in the RTW process.

Intervention programs are delivered to patients with MSDs, CMDs, or a combination of both types of disorders.

### Definition of occupational rehabilitation

Occupational rehabilitation can be conceptualized as *“a timely, goal-oriented, and planned process, in which various stakeholders collaborate to provide the necessary means to empower patients in their effort to achieve optimal functional capacity, coping skills, independence and participation in working life*” [13]. Professionals, particularly rehabilitation teams, work with patients, employers, and social insurance case managers to obtain a timely and safe RTW [14].

### Complex occupational rehabilitation programs

Complex occupational rehabilitation programs include several components that may act both independently and interdependently. They are usually delivered by interdisciplinary or multidisciplinary teams [15]. According to Costa-Black (2013: 432–437), there are several core components of RTW programs for workers with MSDs. They include cognitive-behavioral approaches, education to promote self-care and pain management, education/advice about activity and work, physical exercise programs, and work ability assessments. Additionally, there is often an interface between the workplace and different stakeholders to make the RTW process as effective as possible. Many of the fundamental components are quite similar in programs aimed at both MSD and mental health conditions [15]. According to Wade, effective rehabilitation depends on an expert multidisciplinary team working within the biopsychosocial model of illness and working collaboratively towards agreed goals in a person-centered process [16]. Additionally, as Waddel and Burton denote, RTW interventions do not simply involve the delivery of healthcare directed at symptoms or biological and psychological factors, but also require to simultaneously overcome occupational obstacles, which is why the employer must be involved in the RTW process [14].

### Effect of occupational rehabilitation programs

Several systematic reviews have shown that complex RTW programs may increase RTW rates and may even be cost-effective [15, 17]. Programs that include a workplace component [18] and the use of an RTW coordinator [18] may be more successful than others.

There are few systematic reviews that include analyses of different effects of interventions on RTW among subgroups of patients. A review that did look for subgroups among low back pain patients showed inconclusive results [8]. However, several single trials have shown that there may be differences in the effects of comprehensive interventions compared with treatment as usual or less complex interventions among subgroups of patients. Patients who may benefit more from comprehensive programs are those with high complexity, poor prognosis or a high degree of problems (psychosocial and physical) (MSDs) [19, 20], those with mental health problems [21] who have been out of employment for a long time [22], those who are distressed and who intend to RTW at baseline despite symptoms [23]; and those with low job satisfaction [24]. Patients who are at risk of losing their job and who have little influence on their work situation [25] and those in need of a new job [26] may also benefit from a comprehensive intervention. In addition, while women with MSDs [27] or chronic widespread pain [28] might benefit from a comprehensive intervention, such an intervention might delay the RTW for men with low back pain [28]. Additionally, patients with mental health problems who receive a complex, multidisciplinary intervention detached from the workplace [29] or an exposure-based RTW [30] may have a more delayed RTW than those who receive ordinary case management.

### Modifiable and nonmodifiable factors in RTW interventions

Identifying obstacles to RTW that are modifiable may help occupational rehabilitation clinicians target their interventions. Achieving change at the individual or occupational level that may influence RTW through different short-term, intermediate, and long-term outcomes. A best-evidence synthesis based on systematic reviews identified several factors across different health conditions at the individual level that may be addressed. These factors included health symptoms, health perceptions, general health, functional disability, pain, fatigue, fear-avoidance beliefs, and a lack of motivation to RTW [31]. Several studies have identified RTW self-efficacy as an important and modifiable predictor for actual RTW [32]. Other identified factors at the individual level are self-reported work ability [33], recovery and RTW expectations [34], and increased self-awareness or self-understanding [35]. Increasing one’s activity level, restoring function, and changing behavior are all stated as intervention goals at the individual level to help people RTW [14].

At the organizational level, clinicians may also address several obstacles to their participants’ RTW process through dialog and cooperation with the workplace. Some of the modifiable risk factors for sick leave and delayed RTW that have been identified are physical and psychological demands, job strain, job or role stress, a lack of supervisory support and social support, bullying, low job satisfaction, low job control, poor leadership quality, and a high effort-reward imbalance [36, 37]. A lack of contact with the employer, poor communication, hostile reactions and judgments, and poor organizational support have also been identified as important obstacles to be addressed [38]. Increasing job autonomy, on the other hand, may increase the RTW self-efficacy of injured workers [39]. Interaction and dialog with stakeholders in the workplace are seen as important for strengthening organizational support and finding effective means to avoid delayed RTW [40]. Employees seeking a new employer may also benefit from interaction and coordination between clinicians and other stakeholders, especially disability case managers [41].

Several nonmodifiable prognostic factors for RTW that may influence work participation and the success of the interventions have also been identified. These factors include older age, lower educational level; lower socioeconomic status; and greater sick leave history [42], comorbidities [43], childhood adversities [44], and other life events [45]. Additionally, employment status and partial sick leave at baseline of the intervention [46] and timing of the intervention may also influence RTW [47].

## Methods/design

### Aims

This study will compare the results of two occupational rehabilitation programs in Norway for employees on sick leave due to MSDs, CMDs, and combined and unspecific conditions. In keeping with the aims of a pragmatic design, the selected outcomes are chosen to be relevant to practice [48]. In this setting, relevant stakeholders are physicians, NAV consultants, patients, practitioners, and clinicians who deliver occupational rehabilitation services. The intention is to inform decisions regarding the selection of efficient and cost-effective interventions that help people RTW and decisions regarding for whom an intervention is beneficial and in which circumstances. The selection of secondary outcomes is intended to identify modifiable factors that clinicians may target in their interventions to increase RTW rates among their patients [31, 36]. Another aim is to study the experience of collaboration between stakeholders to support RTW outcomes.

- The primary aim of the study is to evaluate whether there are any differences between the studied programs in the RTW success rates of the participating patients. Since both rehabilitation programs are complex and comprehensive, we hypothesize that there will be no overall differences in RTW rates but that some subgroups might benefit more from one or the other program. As earlier research has identified, there might be differences among subgroups based on gender; age; socioeconomic status; health complexity or severity; length of sick leave; job satisfaction; RTW expectations; the risk of job loss; and work-related strains such as demanding jobs, low decision latitude, conflicts, and poor support.
  Differences in cost-effectiveness will be evaluated if the programs show differences in RTW rates.
- The second aim of the study is to evaluate whether there are any differences between the programs in improvements in known modifiable obstacles to RTW, such as health and function, work ability, RTW expectations, RTW self-efficacy, and fear avoidance, among the participants.
- The third aim of the study is to examine the delivery and reception of the programs to understand how the interventions produce change. This aim will be achieved by investigating the clinicians’ evaluations of how the programs might work; the participants’ expectations of the program and how they respond to and interact with the interventions; and the effect of contextual factors on intervention mechanisms and outcomes in the RTW process, both at the individual and organizational levels.
- The fourth aim of the study is to explore key stakeholder views and experiences of RTW coordination. Key stakeholders are defined as patients, rehabilitation teams, employers, insurance case managers, general practitioners, and occupational health services.
- Our last two aims are to increase our understanding of any differences and similarities in the success of RTW outcomes for the participating clinics by combining the quantitative results for the primary objective of the study with the qualitative and quantitative results from the process evaluation in the study. An overarching aim is to support interactive learning between researchers and rehabilitation professionals for the development of program structures and practices that improve RTW outcomes for their patients.

The project acronym is STAiR, which is a combination of letters from the names of the two participating clinics. The standard protocol items for clinical trials (SPIRIT) are followed [49].

### Study design

The study uses a mixed-method design, in which outcome evaluation is combined with process evaluation. The purpose of the mixed approach in this study is to elaborate on the quantitative RTW results of the interventions by use of a process evaluation to increase our understanding of the mechanisms involved and the influence of the context in the RTW process. Conducting a process evaluation in addition to the outcome evaluation provides a more detailed understanding that is needed to inform policy and practice in the evaluation of complex interventions [10]. The different research components of the study also require different approaches to address the research questions and achieve the aims of the study. We have chosen a pragmatic approach to evaluate the programs as they are delivered in normal practice to the clinics’ usual patient groups with individually tailored services [48]. This approach allows for variations and flexibility in the delivery of services at the two clinics, turnover among health personnel, and other unforeseen variations during the follow-up period.

To evaluate short-term, intermediate, and long-term outcomes and the influence of context in the RTW process, we combine the use of questionnaires, registry data, and several qualitative data collection methods. The quantitative strand related to the main outcome in the study is performed in parallel with the strands associated with the process evaluation. The quantitative and qualitative research questions, data collection, and data analysis will be separate in the investigation of the main outcome but will be combined for the conclusion of the study. The design is categorized as a convergent parallel design following Creswell and Plano Clark [50].

This protocol article mainly presents the quantitative strands of the study, including the questionnaires to be used in the process evaluation. The process evaluation is presented in a separate protocol article [51] and is based on a theory-driven and interactive research design, utilizing program evaluation theory and logic analysis inspired by the principles of realist evaluation [52].

### Study setting and context

In Norway, most employees with MSDs and CMDs visit a general practitioner if they need a sick leave note. According to the National Insurance Act, they are entitled to tax-paid sickness benefits if they are incapable of working due to disease, illness, or injury [53]. Sickness benefits are paid from the first day of absence for a period of up to 52 weeks, usually at the same level as employment income. After the sick leave period, work assessment allowances or disability pensions may be granted if work ability is reduced by 50% or more. The allowances are 2/3 or less of the employment income, with a maximum duration of 3 years. Physicians may refer sick-listed employees to primary care or complex occupational rehabilitation interventions in secondary and tertiary healthcare to facilitate RTW. Complex rehabilitation programs are offered to a small number of persons who need a service that goes beyond what is delivered in primary care. The nature of these persons’ illness, the length of time they have been off work, and their circumstances at work or home often make their needs more complex [4].

### Study population

The study population consists of employees who are referred to either of the two occupational rehabilitation clinics by their practitioners. Those with musculoskeletal disorders and common mental disorders and combined and unspecific conditions will be included. See Table 1 for additional information.

### Recruitment

**Fig. 1:**
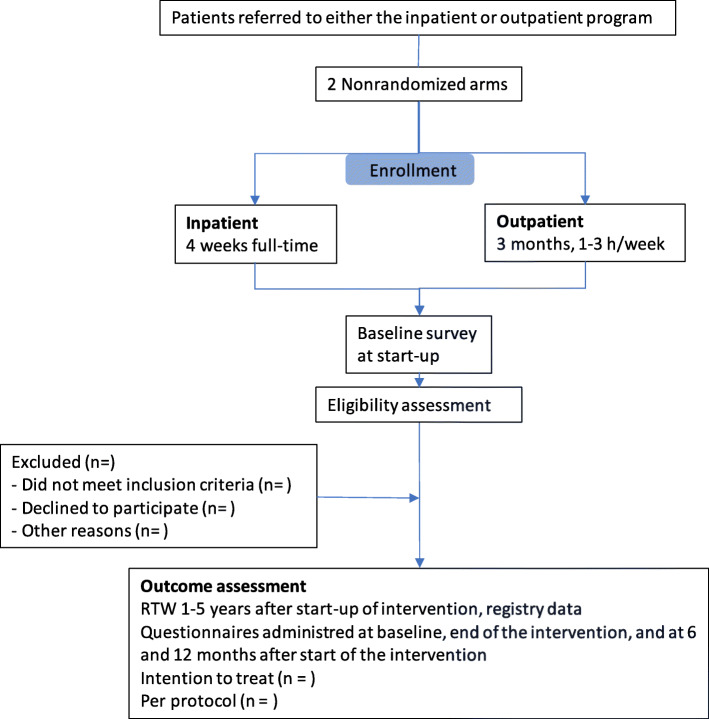
Flow chart of the design of the study

All patients who are referred to either of the two participating clinics for occupational rehabilitation and are approved by the admission team at the clinics will be invited to participate in the study. The patients will receive written information about the study before/when they arrive at the clinics. The provision of supplemental information and the recruitment and collection of the signed agreement forms will be organized by the clinics during the first week of the program. A project assistant will administer the questionnaires and any other follow-up. A flow chart is shown in Fig. 1.

**Table 1:** Inclusion and exclusion criteria

Inclusion criteria	Exclusion criteria
• Employees with MSDs, CMDs, or a combination. They may have additional disorders • Age 18 to 60 years • Sick leave for at least 6 weeks, either in one continuous period or several periods during the last 12 months, related to actual health complaints • Sufficient Norwegian language skills to fill out questionnaires	• Severe psychological disorders (e.g., schizophrenia and other psychotic disorders, bipolar disorder, or personality disorders) • Substance addiction • Pregnancy • More than two years out of work

### Interventions

The participants recruited for the project will receive treatment either in an outpatient occupational rehabilitation clinic at the Hospital of Telemark in Porsgrunn or in an inpatient occupational rehabilitation clinic in Rauland. The clinics are located in secondary and tertiary healthcare in Norway. The interventions will be delivered ‘as usual’ at both clinics by interdisciplinary teams. The components of both interventions may be characterized as 1) *a biopsychosocial component* incorporating individual or group-based cognitive-behavioral approaches, physical exercise, and education; 2) *a work-related component* including the assessment of work ability and readiness for RTW, goal setting, the discussion of rights and responsibilities as an employee on sick leave, efforts to increase RTW self-efficacy and communication with other stakeholders; and 3) *an interdisciplinary component.* Please refer to the process evaluation protocol for more details regarding the conceptual model of occupational rehabilitation and contextual dynamics involved [51]. The inpatient program is more standardized, group-based, and intensive than the outpatient program. The outpatient program, on the other hand, is more individually tailored and has closer collaboration with the workplace and the local community than the inpatient clinic.

#### The program at the outpatient clinic

Two interdisciplinary teams are working at the clinic. Each team consists of a physician, physiotherapist/psychomotor physiotherapist, psychologist, occupational work consultants, and a team coordinator. The program has a duration of 3 months. Four to six new patients arrive each week. On the first day at the clinic, the whole team has individual dialogs with the patients to map their resources, barriers, and possibilities for work [54]. Then, the team discusses the information they have obtained and plans the goalsetting meeting with the patient 2 days later. The patient prepares for the same meeting by writing down their short- and long-term goals for RTW. They also color a visual tool (in the shape of a shoe) that is used to identify their challenges and resources in different areas, such as work, body, self and family, and finances. At the meeting, two representatives from the team and the patient discuss and agree upon an individualized rehabilitation plan. The plan may include individual therapeutic conversations with a psychologist, nurse, or physician. The patients may also receive a course held by the psychologist, covering themes such as anxiety, depression, and stress management, which is organized as education and group conversations. The plan often includes physiotherapy given by a physiotherapist with specialist competence in basic body awareness or psychomotor physiotherapy. The treatment is given individually or in groups. If the patient will benefit from follow-up by health interventions in his or her local community, e.g., due to travel distances, this is also taken into consideration when creating the plan. Each patient has a coordinator who follows up with the patient and coordinates dialogs with the patient’s employer, NAV consultant, or others during the program. Counseling by phone is given when needed. After 3 months, the coordinator has a closing conversation with the patient, usually together with one of the clinicians. In the evaluation made by Kvaal and colleagues [54], most of the patients had one or more individual conversation(s) with one or more team members during follow-up, and the patients had an average of four conversations, with a range between 0 and 14. Approximately half of the patients participated in sessions held by physiotherapists, and one-third of the patients participated in the course held by the psychologist. The typical intensity is 1–2 sessions/week. Communication with the patient’s employer and NAV consultant is often done more than once during the follow-up. It is usually done by phone but is also done through meetings at the clinic or the workplace. For more details regarding the intensity of the intervention, see Table 2.

**Table 2:** Programs at the inpatient and outpatient occupational rehabilitation clinics

Program	Outpatient clinic	Hours	Inpatient clinic	Hours
Duration	3 months	1–3 h/ week	4 weeks	36 h/ week
Mapping process and work ability assessment	Interdisciplinary assessment(questionnaire, interviews, and team discussions)		Interdisciplinary assessment(questionnaire, interviews, and team discussions)	
Cognitive-behavioral approaches	Individual therapeutic conversation and/or treatmentCounseling by phone when needed	1–10 h 0–20 h	Individual therapeutic conversation Group activities	4–15 h 2–4 h
Development of an RTW plan	Patient and team, + individual	1 h	Group process, + individual	6 h
Education to promote self-care and pain management	Education in groups according to individual needs	2 h × 4	Education in groups	7 h
Education/advice about activity and work	Individual counseling with a work consultant	> 1 h	Individual counseling with a work consultant Education in groups with a work consultant	> 1 h 1 h
Exercise program	Psychomotor physiotherapy if needed, one to one or/and in groups	1–10 h	Group activities (gym, water exercise, psychomotor physiotherapy, climbing, Nordic walking, and other outdoor activities). Half of the group can practice riding (75 min × 5)Organized leisure activities and own activities	43 h Varies
Interface with the workplace	Communication via phone and/or meetings. Workplace visits if needed	1–4 h	Communication via phoneSeldom meetings or workplace visits	1–2 h
Communication with other stakeholders	Communication via phone and/or meetings (GP or other healthcare providers or local NAV consultant)	> 1 h	Communication via phone and/or meetings (GP or other healthcare providers or local NAV consultant)	> 1 h
Interdisciplinary team	Physician, psychologist or conversation therapist, NAV−/work consultant, physiotherapist/psychomotor physiotherapist, and team-coordinator		Physician, psychologist or conversation therapist, work consultant, psychomotor physiotherapist, physical education teacher, and team-coordinator. Additional: nutrition counselor, riding instructor, and recreation instructor	
RTW coordinator	Yes		Yes	

#### The program at the inpatient clinic

Three interdisciplinary teams are working with the RTW program at the clinic. Each team consists of a physician, a physiotherapist/psychomotor physiotherapist, a psychologist or a nurse, an occupational work consultant, and a sports pedagogue. One of them has the role of team coordinator. All teams and their patients also have access to a nutrition counselor, a riding instructor, and recreational instructors. Seventeen patients arrive at the clinic at the same time and stay for four consecutive weeks (36 h/week). The physician reads all the information in the referral papers sent from the patient’s general practitioner. In collaboration with the team, it is decided which team members will perform the first mapping conversation with the patient and who will be the coordinator during the patient’s stay at the clinic. The coordinator is responsible for a weekly meeting with the patient and decides whether the patient needs an individual therapeutic conversation with any of the other team members. The coordinator also contacts the employer and/or NAV. The team coordinates its activities and discusses approaches in regular team meetings. Individual goal setting and RTW planning are organized as a process in six group meetings during the stay. Visual tools are also used at the outpatient clinic to stimulate dialogue about the patient’s challenges and resources in the RTW process. The main part of the program is group-based physical activities such as gym, water exercise, psychomotor physiotherapy, climbing, Nordic walking, and other outdoor activities, which are led by sports pedagogues or physiotherapists. Different team members also give educational sessions in groups. The sessions cover themes such as the connection between bodily and psychological reactions, pain management, physical activity and training, introduction to mindfulness, diet and nutrition, and rights and responsibilities as an employee on sick leave. Communication with the patient’s employer and NAV consultant is usually done once during the stay by phone. For more details regarding the intensity of the intervention, see Table 2.

Description of the core components and intensity of the clinics, adopted from Costa-Black et al. 2013: 432 [15].

### Outcome measurements

#### Primary outcome measures

##### Return to work

*RTW* is our primary outcome measure, and it will be measured in different ways with registry data from NAV.

1. Cumulative number of sickness absence days. In line with Aasdahl and colleagues [55], the primary outcome measure is defined as the cumulative number of sickness absence days. By combining information from the different medical benefits, we will calculate the days on medical benefits (according to a 5-day work week) for every month during follow-up. Time on graded sick leave will be transformed into whole workdays. Days receiving sick-leave payment and work assessment allowance will be adjusted for the employment fraction, including a graded disability pension at inclusion. Any increase in the disability pension during follow-up will be counted as sick leave. The cumulative number of sickness absence days will be calculated at 6, 12, 24, and 60 months after inclusion. The evaluation of any subgroup differences will be based on this outcome.
2. Time to stable return to previous or new work, full or partial, for at least 4 weeks without relapse. Time to RTW will be calculated as the number of calendar days measured from the start of the program until 5 years later.
3. The proportions of workers at work full time or part-time, at baseline and every month after the start of participation in the program for 5 years.
4. The proportion of workers who increase or decrease their work status or who have the same work status as they had at baseline. This outcome will be measured at 3, 6, 12, 24, and 60 months after baseline.
5. Individual shifts between work, sick leave, and other social security benefits. This outcome will be monitored continuously based on registry data collected from 2 years before inclusion until 5 years after inclusion in the study [6].

To increase the external validity of the study, the RTW results for items one and two will also be compared to the results of a matched group drawn from the NAV registry.

#### Secondary outcome measures

*Economic evaluation measures:* An economic evaluation will be performed if there is a statistically significant difference in RTW rates between the programs. The main outcome measure will be the incremental costs for an averted sick leave day in the two intervention groups compared to the reference group and in the inpatient program compared to the outpatient program. Data will be collected both through the administration of questionnaires and the extraction of records directly from the clinics. Health economic data will be collected 1 year after the participants’ initiation into the project. Subgroup analyses will also eventually be conducted.

A cost-utility analysis (CUA) will be performed by using a health-related quality of life questionnaire, the SF-12 version 2 [56], and converting the SF-12 scores to SF-6D scores. The SF-12 consists of 12 questions scored on 3- and 5-point Likert scales.

*Work ability:* Work ability will be measured with three items from the Work Ability Index (WAI) [57]. The participants will estimate their current work ability compared with their lifetime best ability. The score ranges from zero (cannot work at all) to ten (best work ability ever). The experienced work ability related to physical and psychological demands is scored on 5-point Likert scales. Work ability will be measured at all time points.

*Return-to-work self-efficacy (RTWSE):* RTWSE will be measured by the *Return-to-Work Self-Efficacy Scale* (RTWSE-19) [58, 59], which has three subscales: *meeting job demands* [7]*, modifying job tasks* [7] and *communicating needs to coworkers and supervisors* [5]. All items are scored on 10-point Likert scales.

*Fear avoidance for work:* Fear avoidance will be measured with a subscale from the *Fear-Avoidance Belief Questionnaire* [60, 61]. The scale consists of seven items and is scored on a 7-point Likert scale.

*Return to work expectations:* RTW expectations will be measured by one item from the WAI questionnaire: *Expectations of ability to remain in the present profession in 2 years*, with the response categories: *barely, uncertain and quite certain*. We will also use two questions from the *Fear-avoidance for work scale*: *I do not think that I will be back to my normal work within 3 months* and *I do not think that I will ever be able to go back to that work*, both scored on a 7-point Likert scale. We will also include an additional question measuring RTW expectations: *If you expect to return to work after occupational rehabilitation, after how long do you expect to return to work?,* with the response categories: *immediately or within 2 weeks, within 1 month, within 2 months, within 3 months, within 4 months, within 5 months, within 6 months, within 1 year, more than 1 year, never, and don’t know/uncertain*.

*Health and illness perceptions*: Health and illness perceptions will be measured with items from a variety of instruments. *General health* will be measured by one item from the SF-12, scored on a five-point Likert scale. *Subjective health complaints (SHCs)* will be measured with the SHC questionnaire [62]. This scale consists of twenty-nine items scored on a four-point scale from zero (no complaints) to three (serious complaints) and has 5 subscales: *musculoskeletal pain* (8 items), *pseudoneurology* (6 items), *gastrointestinal problems* (8 items), *allergies* (5 items), *and flu* (2 items). We will use the first 3 scales. Complaints will be reported for the last 30 days.

From the *Illness Perception Questionnaire* [63], we will include an open-ended response item asking the patients to list the three most important causal factors for their illness.

#### Additional measures

Personal and work-related conditions will be measured with questionnaires. If the origin of the measure is not mentioned, then the measure is adopted from a Norwegian national survey.

Personal-related questions and background variables include the following:

- Gender, age, education, civil status, economy, life events, physical activity, etc.
- An adapted version of the *Adverse Childhood Experiences* (ACE) questionnaire [64].

Measures of work-related conditions include questions drawn from national surveys regarding the following:

- Profession, industry, full time/part-time work, years employed, rank, type of work, working arrangement, type of contract and work accommodations, and expected type of work after rehabilitation.
- Experience of downsizing or restructuring at work and concern about losing one’s job.
- Functional ability and accommodations at work.
- Work characteristics, autonomy [8], social support [2], conflicts [2], job satisfaction [1], job involvement [1], and work commitment [1]. The measures are obtained from the *General Questionnaire for Psychological and Social Factors at Work* (QPSNordic) [65].
- *Effort-reward imbalance*. The measures are taken from the *Effort-Reward Imbalance scale* [66]. We use seventeen of the original twenty-three items across three scales: *effort, reward,* and *overcommitment.*
- *Organizational support* [67]. We use twelve of the original thirty-six scale items.
- *Evaluation of health care services*. Questions regarding the evaluation of health care services are drawn from *The patient satisfaction questionnaire* developed by the Norwegian Knowledge Centre for Health Services [68]. Questions regarding the use of health care services are adapted from Norwegian national surveys. A new questionnaire was developed to evaluate rehabilitation interventions. Work on program theories at the two clinics, the description of program components, interviews with participants, and research on modifiable factors that can be approached in the interventions inspired the selection of the variables.

An overview of outcome assessments, questionnaire items, and timing are given in Tables 3–4 in the appendix.

### Data collection

Quantitative and qualitative data collection methods will be used.

We will obtain registry data from NAV on work participation, sickness benefits, work assessment allowances, and disability pensions. Data regarding labor participation and employment will be collected from the State Register of Employers and Employees administered by NAV or equivalent data from Statistics Norway. Data on education will be obtained from the National Education Database (NUDB). Information about diagnoses and treatments in specialist healthcare will be collected through the Norwegian Patient Registry (NPR). The participants will be followed from 2 years before inclusion until 5 years after their inclusion in the project via their registry data.

Survey data collection will be performed at baseline, the end of the program, and 6 and 12 months after the start-up of the program.

Data on program components, services, and epicrises will be collected from the clinics. Data associated with the participants’ evaluation of the program and their RTW processes will be collected via a survey, and from a group of approximately 40 participants. These participants were interviewed four times: at the start and end of the program and approximately 6 and 12 months after start-up.

Different methods will be used to collect data from the participating teams and key stakeholders (observations at the clinics, interviews, focus groups, and dialog meetings). For more details on the type of data collection that will be used for the process evaluation, please refer to the STAiR process protocol [51]. An overview of the outcome assessments and timing is given in the appendix.

#### Sample size

Estimation of the sample size is based on the assumption that approximately 50% of the patients will have a stable RTW, full-time or part-time, after 1 year. A two-sided survival analysis with a 5% level of statistical significance and 80% power shows that we will be able to detect a statistically significant difference between the programs at a hazard ratio of 0,73 with a sample size of 600 participants. Due to the lower capacity at the outpatient clinic, 200 participants will be recruited from the outpatient clinic and 400 will be recruited from the inpatient clinic.

### Statistical methods and analysis

Analyses of differences in the results between the two interventions will be undertaken both according to intention-to-treat principles and per protocol. Descriptive statistics and standard statistical tools (such as t-tests and chi-square tests) will be used to compare the baseline measurements of the treatment arms.

### Outcome assessment and evaluation

Cox proportional hazard models will be used to analyze the time until stable/sustainable RTW. Analyses will be performed for both full-time and part-time RTW. If there is an imbalance in any prognostic factors for RTW and this factor is strongly correlated with the RTW outcome, analyses of differences in RTW between the two clinics will be adjusted for prognostic dissimilarities if we have sufficient power [69]. To determine the probability of RTW, sick leave, other benefits, or disability pension, a multistate model can be established [70]. This model can determine whether there are differences in the patterns of RTW if differences in the results are discovered in the overall analyses. Since the primary outcome data are based on complete registry data, missing data should not be a problem.

### Subgroup analyses

Subgroup analyses will be performed for the primary outcome (stable/sustainable RTW). All subgroup analyses will be performed using the same analytical model as that for the primary outcome but will also include the subgroup of interest and a treatment-by-subgroup interaction [69]. Interaction tests will be considered significant at the 5% level.

### Cost-effectiveness/utility/benefit analyses

In evaluating the cost-effectiveness/utility/benefit of the interventions, we will follow the Norwegian guidelines for health economic evaluation [71]. In performing the cost-effectiveness analysis (CEA) for the interventions, we will calculate a single combined measure, the incremental cost-effectiveness ratio (ICER). The ICER will be calculated as the difference in cost between the interventions divided by the difference in RTW outcomes. Second, the cost-utility analysis (CUA) will be performed with the SF-12 scores, which will be converted into SF-6D health utility scores as suggested by Brazier and colleagues [72]. The incremental cost-utility ratio will be calculated as the difference between costs for the interventions divided by the difference in the health utility, measured as quality-adjusted life years (QALYs). Any differences between the interventions will also be examined based on a cost-benefit analysis (CBA). In the CBA, the effects of the interventions, e.g., the QALYs and/or the intensity of labor market participation (stable RTW) and/or other health benefits, will be converted into Norwegian kroner.

## Discussion

Occupational rehabilitation programs are complex and are provided in unpredictable, open contexts, which may result in differences in achieved outcomes and variation in effects between subgroups of patients [10]. Mixed-method research draws upon the strength of both quantitative and qualitative approaches and is seen as a powerful tool for investigating complex processes and outcomes in health services research [73]. Combining these methods increases our possibility of understanding “what works” and why [74]. A process evaluation is seen as imperative for understanding the context of the participants, the setting, and the processes in which the results occur to make informed judgments about programs and their applicability to different settings and under different conditions [75]. Pragmatic approaches are also valued since they focus on real-world applications of interventions and are seen as better suited to provide a foundation for informed decisions among alternative treatments for patients, clinicians, and third-party funders [76].

There is still too little knowledge of the effectiveness and cost-effectiveness of inpatient and outpatient occupational rehabilitation programs for RTW among employees on sickness absences due to MSDs and CMDs.

### Strengths of the study

This study is one of the very few studies in Norway to investigate the results of occupational rehabilitation programs on RTW for patients with MSDs, CMDs, or unspecific problems. The longitudinal follow-up and a mixed-method approach with the use of registry data, questionnaires, and process evaluation are strengths of the study. The use of national registry data will eliminate recall bias and increase the quality and reliability of the data on the primary outcome. The use of qualitative data may open ‘the black box’ of the interventions and allow the description of how the clinicians, the patients, and the different stakeholders think the intervention might have worked and which mechanisms might have contributed to the results. Since both the selected patient groups and the delivery of the two programs are as close to normal practice as possible, the external validity of the results is also expected to be strengthened. Additionally, the broad approach of using different research questions and selecting methods with both explanatory and exploratory aims may generate new insight and understanding regarding the content, delivery, and reception of occupational rehabilitation programs; the RTW process; and ‘what works’ and why.

### Limitations of the study

A design with a randomized clinical trial is the preferred design to examine the study questions. This project started as a randomized clinical trial, but due to recruitment problems, it was not feasible to carry through. We stopped recruiting when we had reached approximately 80 participants and decided to change the design. These two randomized arms will be analyzed and reported separately as a pilot study. We do not have enough power in the statistical analyzes to evaluate whether there are any significant differences in effect between the two interventions.

With the two nonrandomized arms described in this article, we will not be able to evaluate whether there are any significant differences in effect between the two interventions, and we will not be able to eliminate the bias associated with differences in the referral of patients to the two clinics. However, interviews with nearly 40 participants, 4 times during a year from the randomized part of the study, will support us with valuable information regarding their reception of the interventions and mechanisms that influence their RTW process.

We know from interviews with participants that there is a bias associated with the referral of patients to the clinics due to patient preferences and possibilities. Some prefer the outpatient program because they can ensure their ability to fulfill family or work obligations or because they are not able to stay away from home for several weeks. On the other hand, some prefer the inpatient program because they can disconnect from family or work for a period.

Those who agree to participate in the study and those who decline may also differ in many unpredictable ways that we are not able to address with the current study design. However, we will look for any selection bias by comparing the demographic statistics and known predictors of RTW among those who agree to participate. The RTW rates in the study will also be compared with national RTW data from an established quality registry for occupational rehabilitation. We also plan to draw a matched group from the NAV registry representing those who receive treatment as usual and to compare RTW rates for this group with the results from the participating clinics.

The study intends to reveal whether there is a difference between the clinics regarding success in RTW rates for different patient groups. When multiple subgroup analyses are performed, the probability of a false-positive finding can be substantial, but when properly planned, reported, and interpreted, they can provide valuable information [77].

### Impact of the study findings

The study aims to inform general practitioners, NAV consultants, and occupational rehabilitation professionals about ‘what works’ and for whom interventions work in occupational rehabilitation, increasing their ability to make informed decisions in the selection of treatment and to develop interventions. With the use of a pragmatic approach and a process evaluation alongside the trial, the transferability of knowledge and possibilities for implementing effective approaches in the network of occupational rehabilitation clinics also increase.

## Data Availability

The datasets generated during the current study will be available upon request from Monica Eftedal, monica.eftedal@air.no. The participants will provide their consent to participate in the STAiR study but not to share their data with other parties. We expect that all data from questionnaires, registry data, and interviews will be available in 2027. To obtain the data, researchers must present research project approval from a Norwegian Regional Committee for Medical and Health Research Ethics or others. To obtain registry data on sick leave, a separate application must be sent to the Norwegian work and welfare administration. Anonymous data can be obtained by communication with the corresponding author.

## Appendix

**Table 3:** Overview of questionnaire items and timing

Assessments	T0	T1	T2	T3
**Individual factors**				
Gender, age, education, marital status, children, demanding caregiver tasks, family economy, life events	X			
Work and benefit status	X	X	X	X
Changes in work and benefit status since start of rehabilitation			X	X
Subjective health complaints (SHC)	X			
SF-12	X	X	X	X
Health and illness perceptions, cause of complaints	X			
Physical activity	X		X	
**Work-related factors**				
Profession, Employment, hours of employment, working time arrangement, work tenure, type of employment (private/public, permanent/temporary), size enterprise	X			
Experienced downsizing, concerns about the job	X			
Accomodations at the workplace	X			
Employer/Colleagues respect functional ability	X			
Expected/plans for work/ work tasks after rehabilitation	X	X		
Job involvement, work commitment	X			
Work ability	X	X	X	X
Effort, reward imbalance (ERI)	X			
Work autonomy, support, conflicts (QPS Nordic)	X			
Organizational support	X			
Job satisfaction	X			
Planning change of employer	X		X	X
FABQ-Work	X	X		
RTW self-efficacy	X	X		
Important factors for RTW (21 items)		X		
RTW expectation	X	X	X	X
**Health Services**				
Evaluation of rehabilitation; improvement health and function		X	X	
Evaluation of rehabilitation; change in self-understanding, increased self-efficacy, motivation for change; improvement work ability, rehabilitation team; program components, collaboration stakeholders		X		
Use of health services	X		X	X

T0 = Enrollment; T1 = End of rehabilitation program; T2 = 6 months; T3 = 12 months

**Table 4:** Overview of outcome assesments

	T0	T1	T2	T3	T4	T5
**Return to work:**						
Cumulated number of sickness days (registry)	X		X	X	X	X
Stable RTW (registry)			X	X	X	X
Proportion of workers full time, part time (registry) (monthly)	X	X	X	X	X	X
Changes work and benefit status (registry) (continuous)	X	X	X	X	X	X
Work and benefit status (questionnaire)	X	X	X	X		
Patient reported changes in work and benefit status		X	X	X		
since start of rehabilitation (questionnaire)			X	X		
**Economic evaluation**						
Incremental costs for an averted sick leave day from a societal perspective				X		
Cost-utility analysis (CUA)				X		
**Modifiable factors at the clinical level**						
General health	X	X	X	X		
Life satisfaction (SF-12)	X	X	X	X		
Work ability	X	X	X	X		
FABQ-Work	X	X				
RTW self-efficacy	X	X				
RTW expectation	X	X	X	X		
Improvement health and function due to program		X	X			
Change in self-understanding due to program		X	X			
Change in self-efficacy due to program		X				
Motivation for change due to program		X				
Improvement work ability due to program		X				
Goal-setting for RTW		X	X			
**Evaluation of rehabilitation program**						
Benefit from the program as a whole		X				
Benefit from the program components		X				
Interaction with rehabilitation team-members		X				
Collaboration with stakeholders during the program		X				
Planned follow-up after the program		X				
What facilitates and hinder the RTW-process (qualitative)	X	X	X	X		

T0 = Enrollment; T1 = End of rehabilitation program; T2 = 6 months; T3 = 1 year; T4 = 2 years; T5 = 5 years

## Abbreviations

RTW: Return to work
CMD: Common mental disorder
MSD: Musculoskeletal disorder
